# Myocardial extracellular volume fraction (ECV) measurements based on T1-mapping in healthy monozygotic twins - another similarity or rather disagreement in twins?

**DOI:** 10.1186/1532-429X-17-S1-Q31

**Published:** 2015-02-03

**Authors:** Anca R Florian, Anna Ludwig, Sabine Rösch, Elke Schaeffeler, Matthias Schwab, Udo Sechtem, Ali Yilmaz

**Affiliations:** 1 Cardiology, Uniklinikum Muenster, Muenster, Germany; 2 Cardiology, Robert Bosch Hospital, Stuttgart, Germany; 3 Institute for clinical pharmacology, Stuttgart, Germany

## Background

The T1-mapping MOLLI technique has already proved to be a robust and reproducible technique for myocardial extracellular volume fraction (ECV) estimation in both healthy individuals and patients with different cardiomyopathies. Monozygotic twins share the same DNA information and frequently grow up in a similar environment. However, epigenetic as well as environmental factors may lead to major differences in clinical phenotype, organ function as well as character and behaviour. Therefore, monozygotic twins provide a very interesting and attractive research model regarding specific organ structure, metabolism and function.

With this study, we aimed at evaluating possible ECV measurement variations within pairs of healthy monozygotic twins.

## Methods

Fourteen pairs of healthy monozygotic twins (N=28; 21% male; mean age 25±5 years) underwent cardiovascular magnetic resonance (CMR) studies including pre- and post-contrast T1-mapping (MOLLI) and late-gadolinium-enhancement (LGE)-imaging at a 1.5-T scanner (Siemens Aera). Global ECV was calculated for each participant in a mid-ventricular short axis slice. The twins from each pair were assigned randomly to one of two following groups: TWINS A and B. In order to assess variability of ECV measurements, global ECV was measured a second time in TWINS A. Delta ECV was calculated between TWINS in each pair (Delta TWINS) and between the initial and the second measurement in TWINS A (Delta Variability). The respective analyses were performed blinded.

## Results

Mean LV end-diastolic volume was 143±35 mL and ejection fraction was 63±6%. No significant differences in LV volumes, mass and ejection fraction were noted between TWINS A and B. None of the TWINS exhibited any presence of LGE. Mean global ECV was 27±3%. In the paired analysis, there was no significant difference in global ECV between TWINS A and B (27±3% vs. 27±3%, p=0.26) or between the two measurements in TWINS A (27±3% vs. 27±3%, p=0.63). Strong correlations were detected in global ECV within the pairs of TWINS A and B (Pearson R = +0.70, p=0.005) and between the two measurements in TWINS A (Pearson R = +0.64, p=0.015). No significant difference was noted between Delta TWINS and Delta Variability (0.8±2.5 vs. -0.4±2.7, p=0.26).

## Conclusions

ECV measurements in pairs of healthy monozygotic twins demonstrated similar global ECV values that did not vary more than the expected variability of measurement. Moreover, ECV measurements showed a very good reproducibility.

## Funding

None.

